# Tacky-Free Polyurethanes Pressure-Sensitive Adhesives by Molecular-Weight and HDI Trimer Design

**DOI:** 10.3390/ma14092164

**Published:** 2021-04-23

**Authors:** Ji-Hong Bae, Jong Chan Won, Won Bin Lim, Byeong Joo Kim, Ju Hong Lee, Jin Gyu Min, Min Ji Seo, Young Hyun Mo, PilHo Huh

**Affiliations:** Department of Polymer Science and Engineering, Pusan National University, Busan 609-735, Korea; jihong.bae@pusan.ac.kr (J.-H.B.); jcwon@pusan.ac.kr (J.C.W.); freewonbin@nate.com (W.B.L.); mielstylo@pusan.ac.kr (B.J.K.); dlwnghd15@pusan.ac.kr (J.H.L.); jg_min0629@naver.com (J.G.M.); mj1029love@naver.com (M.J.S.); yhmo987@pusan.ac.kr (Y.H.M.)

**Keywords:** polymer, polyurethane, pressure-sensitive adhesives, tacky-free, peel, adhesion

## Abstract

Polyurethane pressure-sensitive adhesives (PU-PSAs) with satisfactory tack, cohesion, and removability were newly developed through the synthetic process by reacting methylene diisocyanate, poly(ethylene glycol) (PEG), and a 1,4-butanediol chain extender based on the different HDI/HDI trimer ratios. The sticking properties of PU-PSAs depended on both the HDI/HDI trimer ratio and crosslinking-agent composition in the formulation. The molecular weight (MW) dependence of adhesion in PU-PSA was observed in the range of 1000 < Mn < 3000, suggesting that the increase in MW limits the pressure-sensitive adhesion of these samples. The differences in the crosslinking-density significantly affected the cohesion, adhesion, and tack in PU-PSA. The formulation of 50 wt.% 600PEG and 50 wt.% crosslinking-agent and an HDI/HDI trimer ratio of 1.0 led to the optimal balance between the adhesion and cohesion properties owing to the sufficient tack, high 180-peel strength, and good cohesion.

## 1. Introduction

Pressure-sensitive adhesives (PSAs) are viscoelastic materials that can bond to different substrates physically after applying very slight pressure for a short time [1,2,3]. PSAs must form viscoelastic bonds that are aggressively and permanently tacky and can be removed cleanly without leaving any residual substance. Regarding the viscoelastic properties of PSA, the viscous portion can be assigned to adequate bonding-wetting ability, and the elastic portion can be assigned to the clean debonding ability [4,5,6]. PSAs have been applied in various adhesive tapes, labels, or repositionable adhesives in the food, pharmaceutical, security packaging, and automotive industries. They are also promising candidates for various medical products, such as patches, surgical bandages, biomedical electrodes, and medical plasters [7]. The three main viscoelastic properties of PSAs are tack, which is the immediate bonding ability to allow wetting and adhere quickly on various substrates; shear adhesion, which is the resistance-ability to resist flow under creep; and peel strength, which is the debonding ability to allow easy removal by peeling without leaving adhesive residue [8].

PSAs can be classified into several types according to their composition and manufacturing process, such as polyurethane-based PSAs, acrylic PSAs, polysiloxane-based PSAs, and polyisobutylene-based PSAs. The composition of polyurethane-based PSAs is relatively simple, which involves three major structures: soft-segments related to glass transition to provide tackiness, hard-segments related to the melt transition to provide cohesion, and networks for adhesion via crosslinkers. Polyurethane pressure-sensitive adhesives (PU-PSAs) have attracted considerable interest in the industrial/consumer industries because of their good performance, environmental friendliness, and easy practical application. PU-PSAs show adequate resistance to heat and solvents and have better low temperature and hydrophilic performance than the acrylic- or rubber-PSAs. On the other hand, PU-PSAs exhibit some limitations, such as relatively low tackiness, low peel adhesive strength, and high moisture permeability. To overcome these problems, the molecular-structures of PU-PSA have been designed by changing the HDI/HDI trimer ratio, curing-agent composition, and the interactions of hard/soft segments. Fuensanta et al. has operated PU-PSA using polyethers and polypropylene glycols (PPGs) of different molecular weights (MWs) [9,10,11,12,13]. Baron et al. and Akram et al. reported the synthesis and characterization of grafted PU by the introduction of crystallizable fragments and mixtures of PPGs with different MWs [14,15].

In this study, the molecular formulations of PU-PSAs were designed by controlling the MWs of polyol and the composition of curing-agent, which can be tuned mainly by the ratios of hard/soft segments and HDI/HDI trimer in the synthesis of PU-PSAs. This study focused on the effects of a combination with different polyol MWs and curing-agent contents based on the molecular design to achieve a good balance between adhesion and cohesion. The adhesive properties and morphological structure of PU-PSAs, including tackiness and peel strength, were evaluated using a universal test machine (UTM), field emission scanning electron microscopy (FE-SEM), and atomic force microscopy (AFM).

## 2. Experimental

### 2.1. Materials

Polyethene glycol (PEG, Mn = 600 and 2000 g/mol, Tokyo Chemical Industry Co., Ltd., Tokyo, Japan) was used as a first polyol. Hexamethylene diisocyanate (HDI, Tokyo Chemical Industry Co., Ltd., Tokyo, Japan) and HDI-based polyisocyanate trimer (Aekyung Chemical Co., Ltd., Seoul, Korea) were used as the aliphatic diisocyanate and co-diisocyanate, respectively. 1,4-butanediol (1,4-BD, Junsei Chemical Co., Ltd., Tokyo, Ja-pan) and polyoxyalkylene polyol (KPX Chemicals, Seoul, Korea) were used as the second polyol. The polyols were melted and dried at 80 °C in a vacuum oven for three hours before use.

### 2.2. Synthesis of Polyurethane Pressure-Sensitive Adhesive (PU-PSA)

**Scheme 1:**
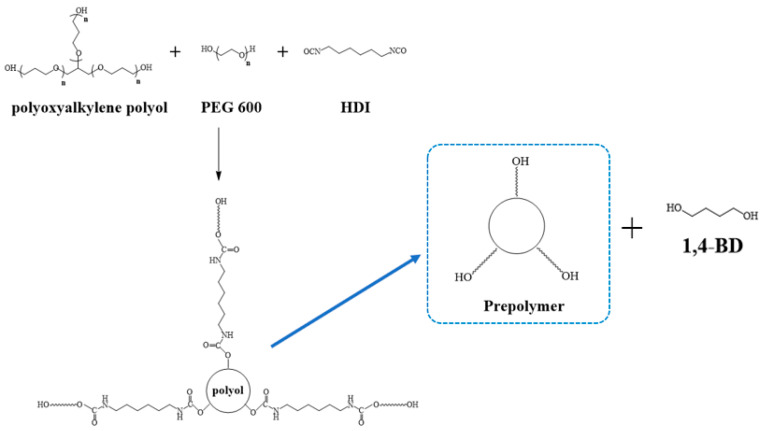
Synthetic procedure to form the PU-PSA prepolymer.

**Scheme 2:**
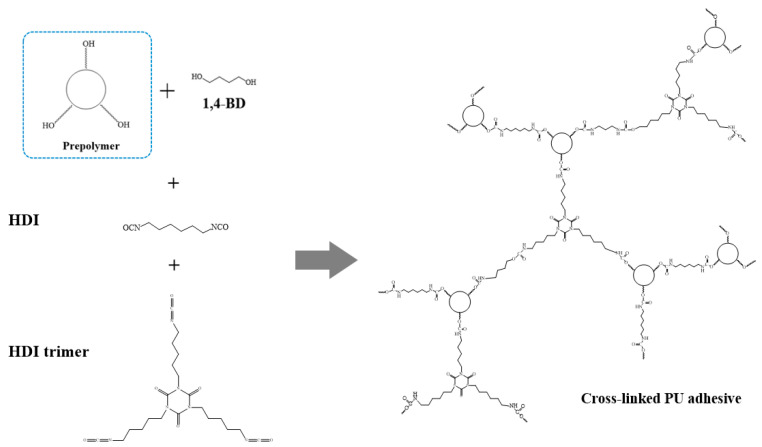
Schematic progress diagram to crosslink the PU-PSA.

A polyurethane pressure-sensitive adhesive (PU-PSA) was synthesized using the prepolymer method in a 500 mL four-neck round-bottom glass reactor under N2 purging conditions. 7.00 g of HDI was melted at 70 ℃, and 3.33 g of PEG + 20.00 g of polyoxyalkylene polyol was added under mechanical stirring of 250 rpm. The chain extender (1.00 g of 1,4-BD) was added sequentially, and the mixture is stirred at 70 °C and 80 rpm for one hour. To prepare the PU-PSA, 10.00 wt.% of HDI and HDI-based polyisocyanate trimer mixture were poured into the PEG + HDI + polyoxyalkylene prepolymer and allowed to cure for five minutes at room temperature. Scheme 1 and Scheme 2 present the synthetic and curing procedures of PU-PSA, respectively.

### 2.3. Characterization

The Fourier transform infrared (FT-IR, Tensor 27, Bruker Optik GmbH, Erlinger, Germany) spectra of PEG + HDI + polyoxyalkylene prepolymer and the cured PU-PSA were obtained at a resolution of 4 cm^−1^ over the spectral range from 400 to 4000 cm^−1^. The adhesion and cohesion properties of PU-PSA were carried out in an Instron 4411 universal testing machine (UTM) based on the PU-PSA strips with dimensions of 30 mm × 300 mm × 0.5 mm. Five samples were tested and averaged for each measurement. The morphological properties of the cured PU-PSAs were performed using FE-SEM and AFM.

## 3. Results and Discussion

**Figure 1:**
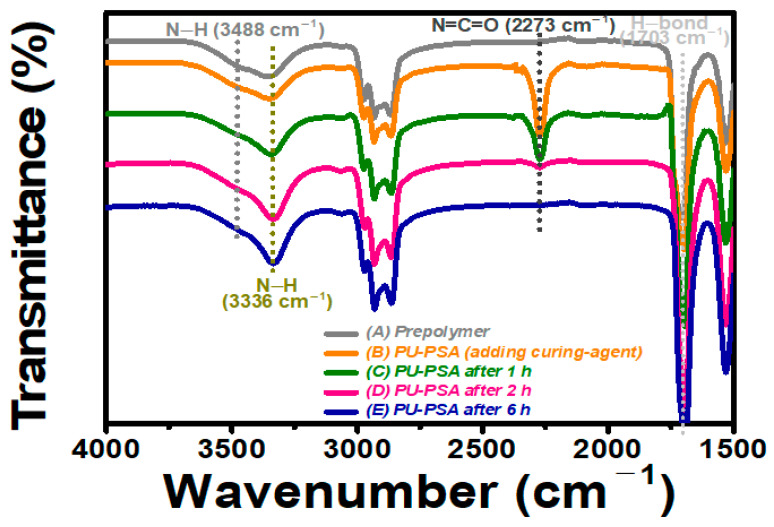
FT-IR spectra of PU-PSA synthesized by a two-step reaction.

The preparation of PU-PSA by the addition of a curing-agent was confirmed by FT-IR spectroscopy. Figure 1 shows the FT-IR spectra in a synthetic procedure of PU-PSA. The two absorption peaks at 3488 and 3336 cm^−1^ were assigned to the asymmetric and symmetric N–H stretching of the urethane group, and the intense band at 1598 cm^−1^ was assigned to the in-plane N–H bending of the hard segments. As shown in Figure 1, the characteristic absorption peaks for N=C=O were observed at 2274 cm^−1^, which strongly appears in (B), slightly vanishes in (C), almost vanishes in (D), and vanishes completely in (E) due to the end-capping and entire reacting of N=C=O. FT-IR region of 1670–1750 cm^−1^ can be assigned to C=O stretching, which may be related to the hydrogen-bonded C=O (hydrogen bonding between C=O and N–H) of urethane linkage and the absorbance of 1720 cm^−1^ may be related to free C=O group [16,17,18]. The contribution of H-bonding by urethane groups increased with increasing the hard segment, as shown in Figure 1. Hence, all reactants after adding the curing-agent react completely to produce the PU-PSA.

**Figure 2:**
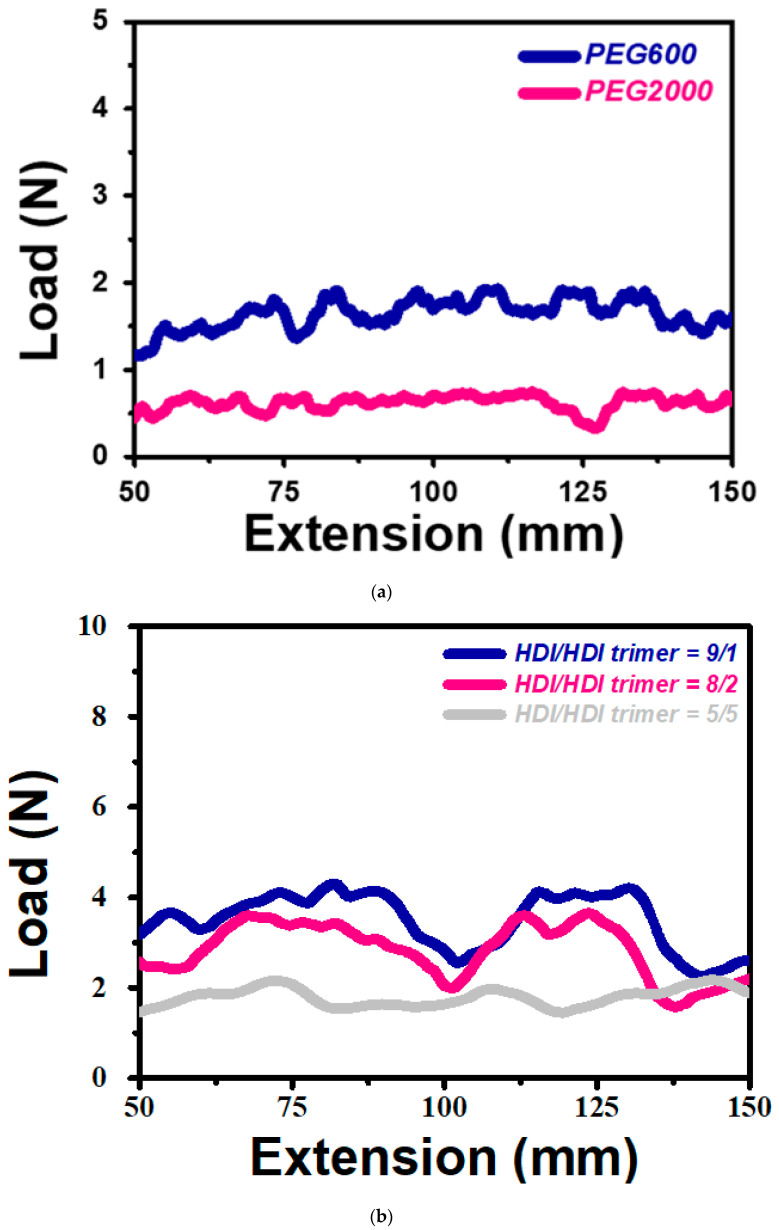
(**a**) 180° peel strength patterns of PU-PAS as a function of PEG MW and (**b**) bonding strength patterns between PU-PSA and glass-adherend by different HDI/HDI trimer ratios.

The effects of the molecular weight (MW) on the sticking-strength were studied to tune the holding power under different application conditions. Figure 2a shows the peel strength patterns of PU-PSA depending on the different PEG MWs during the 180° peel test. As shown in Figure 2a, both curves by different MWs of PEG 600 and 2000 showed that a stable sticking-pattern within the peel strength occurred in the range from 50 to 150 mm extension. A low MW value can enhance the sticking-strength of PU-PSA because of the larger crosslinking points and crosslinking density. This could also be induced by increased H-bonding. Figure 2b presents the bonding strength that forms instantly between the PU-PSA and glass adherend as a function of the HDI/HDI trimer ratio. After the HDI/HDI trimer ratio was changed from 9/1 and 8/2 to 5/5, the peel force decreased to 2.89 Ncm^−1^ and 1.48 Ncm^−1^, respectively. The strong bonding strength may lead to the generation of rough surfaces that occur during the attachment to and detachment from the adherend. The tacky properties of PU-PSA are dependent on the HDI/HDI trimer to resin-formulation ratio, as a high HDI content produces a relatively strong but sticky adhesive. In contrast, a high HDI trimer content generates a relatively soft but non-sticky adhesive.

**Figure 3:**
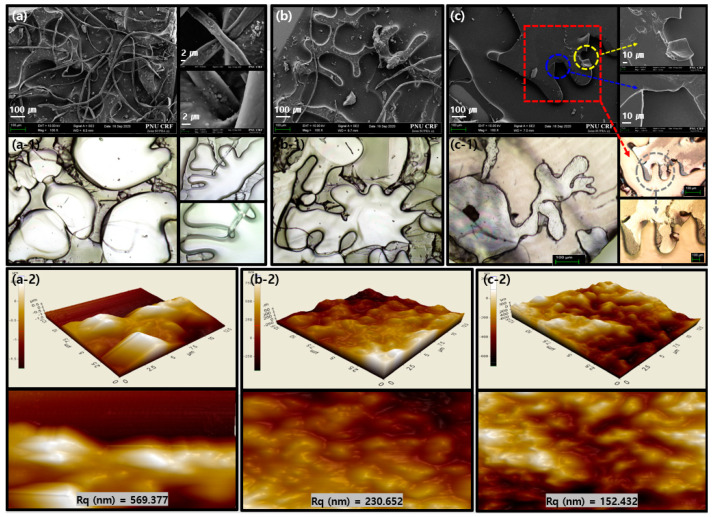
FE-SEM, optical microscopy and AFM images of PU-PSA interfaces after detachment to the adherend; (**a**), (**a-1**), (**a-2**): HDI/HDI trimer = 9/1, (**b**), (**b-1**), (**b-2**): HDI/HDI trimer = 8/2 and (**c**), (**c-1**), (**c-2**): HDI/HDI trimer = 5/5.

The topographical information at the surface after the 90° peel test was measured to confirm the effect of the HDI/HDI trimer ratio on the tacky-ability of PU-PSA. Figure 3 presents FE-SEM, optical microscopy, and AFM images of the broken-interface between PU-PSA and glass substrate after an attach/detach cycle. Figure 3a–c and Figure 3a-1–c-1 show the presence of torn and smooth PSA layers, which are distributed randomly throughout the broken-interface of the PU-PSA matrix. With increasing HDI trimer content, the PSA domains are loosely attached and detached cleanly without any PSA debris. This can be explained by the 5/5 control of the HDI/HDI trimer optimizing the interaction and aggregation of PSA molecular chains to remove the PSA neatly attached to the glass substrate. In other words, the wetting-ability between PU-PSA and the substrate leads to effective interface bonding. In the AFM images of Figure 3a-2–c-2, the detached PU-PSA with the 9/1 HDI/HDI trimer exhibits the roughest surface compared to the others, which indicates a great degree of agglomeration. Consequently, the HDI/HDI trimer dependence of tacky-ability was observed over the range, 569.3 nm < roughness < 152.5 nm, indicating that a higher extent of the HDI trimer among the molecular chains involves more interactions and aggregation of the PU-PSA matrix.

## 4. Conclusions

A novel PU-PSA was formulated based on polyurethane to determine the optimal bonding strength that forms instantly on a glass adherend. The decreasing PEG MW leads to an increase in crosslinking points, causing a higher degree of crosslinking, which also may benefit H-bonding of the molecular chains. The increase in HDI trimer content leads to both a better residue level and poorer peel strength. This phenomenon can be attributed to the increased cohesive strength between the hard/soft segments. The resulting PU-PSA can be an excellent candidate material for use as a completely removable PSA in labels and medical patches.

## Data Availability

The data presented in this study are available on request from the corresponding author.
